# Effectiveness of nasal irrigation devices: a Thai multicentre survey

**DOI:** 10.7717/peerj.7000

**Published:** 2019-05-27

**Authors:** Patorn Piromchai, Charoiboon Puvatanond, Virat Kirtsreesakul, Saisawat Chaiyasate, Sanguansak Thanaviratananich

**Affiliations:** 1 Department of Otorhinolaryngology, Faculty of Medicine, Khon Kaen University, Khon Kaen, Thailand; 2 Department of Otolaryngology, Faculty of Medicine, Prince of Songkla University, Hat Yai, Songkhla, Thailand; 3 Department of Otolaryngology, Faculty of Medicine, Chiang Mai University, Chiang Mai, Thailand

**Keywords:** Devices, Sinusitis, Allergic rhinitis, Cold, Nasal irrigation

## Abstract

**Background:**

Nasal irrigation is widely used as an adjunctive treatment for nasal diseases. There is little evidence regarding the efficacy of the devices used in this procedure. The objective of this survey was to evaluate the effectiveness of nasal irrigation devices based on the experiences of patients and physicians.

**Methods:**

We conducted a multicentre survey study between November 2017 and October 2018. Physician and patient questionnaires were developed based on the available literature and expert opinion. The physician questionnaire was submitted to the Otolaryngology residents and staff of each centre and their network. The physicians were also asked to distribute the patient questionnaire to their patients.

**Results:**

Information regarding 331 devices used by the patients was collected. The mean age of the patients was 45.46 ± 17.19 years (from 5 to 81). Roughly half were male, and half were female (48.6%: 51.4%). Among the high-pressure devices, we found that the high-pressure large-volume nasal irrigation devices yielded significantly higher symptom scores in seven of 12 domains (*p* < 0.05). Among the large-volume devices, we found that the large-volume high-pressure nasal irrigation devices received significantly higher symptom scores in 4 of 12 domains (*p* < 0.05). However, a higher proportion of patients using the large-volume high-pressure devices had retained fluid in the sinuses compared to those using large-volume low-pressure devices (*p* < 0.001).

**Conclusions:**

This survey supports the regular use of nasal irrigation, particularly with large-volume high-pressure devices, as an effective treatment for nasal disease. It may be effective at clearing nasal secretion, improve nasal congestion, decrease post-nasal drip, improve sinus pain or headache, improve taste and smell, and improve sleep quality. It could be used by patients with good compliance and minimal side effects.

## Introduction

Nasal irrigation is a common procedure used to unblock the nose by gently rinsing the nasal and sinus cavities. It facilitates the mechanical removal of mucus (Karadag, 2002; Kurtaran et al., 2003), infective pathogens (Piromchai et al., 2013), inflammatory mediators (Georgitis, 1994; Ponikau et al., 2005), promotes ciliary beat frequency (Boek et al., 2002; Piromchai et al., 2013; Talbot, Herr & Parsons, 1997) and strengthens the antimicrobial and antiviral barrier function (Jantsch et al., 2015; Ramalingam et al., 2018; Ramalingam et al., 2019).

In Thailand, according to the National Statistical Office of Thailand from 2009 to 2017, there were approximately 30 million outpatient visits per year for respiratory tract diseases (National Statistical Office of Thailand, 2019). From our experience, we estimated up to 80 percent of this population have been using the nasal irrigation devices.

According to standard guidelines, nasal irrigation was recommended to use as an add-on therapy for various nasal conditions, such as those laid out in the European Position Paper on Rhinosinusitis and Nasal Polyps (Fokkens et al., 2012), the BSACI guideline for the diagnosis and management of allergic and non-allergic rhinitis (Scadding et al., 2017), and the American Academy of Otolaryngology-Head and Neck Surgery Clinical Practice Guideline in Adult Sinusitis (Rosenfeld et al., 2015).

However, there is no consensus as to the most effective nasal irrigation method. A recent randomised controlled trial found the patients using high-volume low-pressure nasal irrigation device had a better nasal finding score (Salib et al., 2013). Hence, more evidence is needed to support this study.

The Thailand Research Fund has endorsed a program to evaluate the efficacy of nasal saline irrigation in current practice (RSA6080040). One part of this program includes a survey of the effectiveness and safety of nasal saline irrigation. This survey was designed to determine the effectiveness and safety of various nasal irrigation devices from the perspective of patients and physicians based on their previous experience.

## Materials & Methods

### Study design and setting

We conducted this multicentre survey study between November 2017 and October 2018. The questionnaires were distributed at three university hospitals (Khon Kaen University, Chiang Mai University, and Prince of Songkhla University) in different regions of Thailand.

### Questionnaire

Patient and physician questionnaires were developed based on the available literature (Brozek et al., 2017; Chong et al., 2016; Head et al., 2018; King et al., 2015; Shaikh & Wald, 2014) and expert opinion. The patient questionnaire consisted of questions regarding general personal information, devices used, saline solution concentration, and effectiveness scores ranging from 0 to 10 (0 = strongly disagree, 10 = strongly agree) that covered disease severity, convenience of use, learning curve, and device satisfaction. The physician questionnaire asked about the advantages and disadvantages of each device and the most and least effective device based on the physician’s professional and personal experience.

### Participants

The physician questionnaire was distributed to Otolaryngology residents and staff at each centre as well as others in its network. The physicians were also asked to distribute the patient questionnaire to their patients that have been used the nasal irrigation device before. All participants were informed that filling in the questionnaire was considered consent to use the collected data for research. Participation was voluntary.

### Devices

There were various types of nasal irrigation device. We can categorise the device according to the volume of the saline solution used. A high-volume device used more than 100 ml of saline solution to irrigate the nose (Principi & Esposito, 2017). The devices can also further divide according to the pressure of the solution when introducing into the nose. The low-pressure devices using gravitational pressure, or the solution will be expelled from the nose when the pressure is high.

The solution for irrigation can be hypertonicity, isotonicity and hypotonicity. There was limited evidence of whether a specific solution was superior to other solutions according to a recent systematic review (Kanjanawasee et al., 2018). Hypertonic and hypotonic saline may carry greater side effects such as nasal irritation and mucosal cell damage (Baraniuk et al., 1999; Kim et al., 2005).

The devices in this survey were divided into

• Low-volume, low-pressure (e.g., nasal drops)
• Low-volume, high-pressure (e.g., nasal spray)
• High-volume, low-pressure (using gravitational pressure or the saline will be expelled from the nose when the pressure is high; e.g., Neti pot, syringe, bulb)
• High-volume, high-pressure (e.g., syringe with adapter, squeeze bottle).

### Ethical consideration

The research protocol was reviewed and approved by the Khon Kaen University Ethics Committee for Human Research (HE601419). Informed consent was waived due to the nature of this study.

### Statistical Analysis

The sample size was calculated based on a pilot study on ten patients. The mean nasal symptom score was 6.88 ± 2.40 (from 0–10). We expected the variation in the mean score was 5 percent. In order to attain a significance level of 0.5 and power of 90 percent, we determined that a total of 245 devices experience would be required.

Statistical analyses were performed using the SPSS version 20 and Stata version 14. Data were described as either means (for the continuous variables) or frequencies and percentages (for the categorical variables). The normality of the data was evaluated by the quantile–quantile plot. Significant differences between groups were determined using the Student *t*-test or the Mann–Whitney *U* test for continuous variables. The chi-square test or Fisher’s exact test was used to determine whether there was a significant difference between the expected frequencies and the observed frequencies. The multivariate analysis was performed using generalised linear regression model. For all tests, *p* < 0.05 was considered statistically significant.

## Results

### Patient survey

Information regarding 331 devices used by 255 patients was collected. Two hundred fifty-six records were from Khon Kaen University, 75 were from Chiangmai University, and 56 were from Prince of Songkhla University. As the questionnaires were completed and collected in an outpatient clinic setting, the response rate was 100 percent.

The highest proportion of patients performed nasal irrigation for allergic rhinitis (*n* = 109, 43%) and chronic sinusitis (*n* = 81, 32%), followed by post-surgery (*n* = 53, 21%) and common cold (*n* = 31, 12%). Other diseases included acute sinusitis, nasal polyps, nasopharyngeal cancer, sinus cancer, and epistaxis. The mean age of the patients was 45.46 ± 17.19 (SD: Standard deviation) years (from 5 to 81). Roughly half were male, and half were female (48.6:51.4%). Most of the patients did not smoke (*n* = 212, 86.5%).

Of the 331 devices used by patients in this study, 201 were syringes (60.7%), 50 were squeeze bottles (15.1%), 31 were sprays (9.4%), 27 were syringes with nasal adapters (8.2%), 13 were bulbs (3.9%), and nine were Neti pots (2.7%). None of the patients performed nasal saline drop. No patient used hypertonic or hypotonic saline solution.

The duration of device use ranged from 1 to 216 months (mean 23.35 ± 29.32 months). Most of the patients performed nasal irrigation twice a day (51.4%). Patients were mostly taught how to use the devices by physicians (47.4%) and nurses (35.6%). Most patients flexed their necks (72.8%) and did not breathe (65.3%) during irrigation.

### High-pressure devices

High-pressure nasal irrigation devices can be divided into small- and large-volume devices. The high-pressure small-volume nasal irrigation device used in this study was nasal spray (*n* = 31), while the high-pressure large-volume nasal irrigation devices used in this study included syringes with nasal adapters (*n* = 27) and squeeze bottles (*n* = 50).

#### Symptom scores

The high-pressure large-volume nasal irrigation devices received significantly higher scores in seven of the 12 nasal-sympton domains (*p* < 0.05) including overall symptoms (MD (Mean difference) 1.02; 95% CI [0.27–1.7]), nasal congestion (MD 0.95; 95% CI [0.12–1.78]), pain/headache (MD 1.55; 95% CI [0.37–2.72]), post-nasal drip (MD 1.76; 95% CI [0.63–2.90]), improve taste and smell (MD 1.44; 95% CI [0.08–2.79]), clear the secretion (MD 2.05; 95% CI [1.03–3.08]), and improve sleep quality (MD 1.25; 95% CI [0.20–2.30]; Table 1).

**Table 1:** Symptom scores for high-pressure devices.

**Scale (0–10; higher is better)**	**High-pressure devices**		**Mean difference**	***p*-value**	**95% CI**
	**Small-volume(*n* = 31)**	**Large-volume(*n* = 77)**			
1. Improve overall symptom	7.45 ± 2.12	8.46 ± 1.60	1.02	0.008^*^	0.27 to 1.7
2. Improve nasal congestion	7.33 ± 2.32	8.28 ± 1.75	0.95	0.025^*^	0.12 to 1.78
3. Decrease runny nose	7.23 ± 2.27	8.06 ± 1.88	0.82	0.061	−0.04 to 1.69
4. Decrease blowing nose	7.23 ± 2.24	7.97 ± 2.17	0.74	0.123	−0.20 to 1.68
5. Decrease viscosity	7.50 ± 1.84	8.32 ± 1.89	0.82	0.051	−0.01 to 1.64
6. Improve sinus pain/headache	6.26 ± 2.66	7.81 ± 2.07	1.55	0.010^*^	0.37 to 2.72
7. Decrease post-nasal drip	6.13 ± 2.70	7.89 ± 2.23	1.76	0.003^*^	0.63 to 2.90
8. Improve taste and smell	6.35 ± 2.66	7.79 ± 2.60	1.44	0.038^*^	0.08 to 2.79
9. Decrease sneezing	6.83 ± 2.71	7.56 ± 2.31	0.74	0.224	−0.46 to 1.93
10. Decrease cough	6.50 ± 2.79	7.17 ± 2.70	0.68	0.412	−0.96 to 2.31
11. Clear the secretion	6.30 ± 2.74	8.36 ± 1.95	2.05	<0.001^*^	1.03 to 3.08
12. Improve sleep quality	7.1 ± 2.49	8.35 ± 1.88	1.25	0.020^*^	0.20 to 2.30

**Notes.**

* *p* < 0.05.

CI confidence interval

The multivariate analysis using linear regression was performed to evaluate the impact of sex, age, smoking status, frequency of nasal irrigation, and nasal conditions on the overall symptoms score. However, the result indicated these factors did not reach a statistically significant level of impact on the overall symptoms score (*p* < 0.05).

#### Ease of use, learning curve, and satisfaction

Both types of high-pressure nasal irrigation device received excellent scores (mean score more than 7.5, scale 0–10 higher is better). However, the high-pressure large-volume nasal irrigation devices were more likely to be recommended by the patients (MD 0.45; 95% CI [0.26–2.03]) (Table 2).

**Table 2:** Ease of use, learning curve, and satisfaction scores of high-pressure devices.

**Scale (0–10; higher is better)**	**High-pressure devices**		**Mean difference**	***p*-value**	**95% CI**
	**Small-volume(*n* = 31)**	**Large-volume(*n* = 77)**			
1. Ease of use	8.61 ± 1.99	8.83 ± 1.74	0.23	0.567	−0.57 to 1.03
2. Learning curve	8.71 ± 1.84	8.99 ± 1.49	0.27	0.444	−0.43 to 0.97
3. Satisfaction score	8.19 ± 1.96	8.94 ± 1.85	0.76	0.075	−0.08 to 1.60
4. Would recommend the device to others	7.86 ± 2.58	9 ± 1.75	0.45	0.012^*^	0.26 to 2.03

**Notes.**

* *p* < 0.05.

CI confidence interval

In the subgroup analysis, allergic rhinitis patients rated higher satisfaction score in high-pressure large-volume devices (MD 1.21; 95% CI [0.21–2.22]) and would recommend the high-pressure large-volume devices to others (MD 1.63; 95% CI [0.50–2.76]). There was no statistical difference between two groups in other nasal diseases.

#### Adverse events

The most common adverse event in patients using the high-pressure devices was retained fluid in the sinuses (12.9 and 29.9% for small-volume and large-volume high-pressure devices, respectively). There was no statistically significant difference in terms of adverse events between subgroups for each type of device (Table 3).

**Table 3:** Adverse events of high-pressure devices.

	**High-pressure devices**		**Risk difference**	***p*-value**	**95% CI**
	**Small-volume(*n* = 31)**	**Large-volume(*n* = 77)**			
1. Pain/discomfort	2 (6.4%)	1 (1.2%)	−5.5%	0.140	−14.16 to 3.86
2. Retained fluid in sinuses	4 (12.9%)	23 (29.9%)	16.97%	0.066	1.35 to 32.8
3. Epistaxis	0 (0%)	1 (1.3%)	1.30%	0.524	−1.23 to 3.83
4. Headache	0 (0%)	1 (1.3%)	1.30%	0.524	−1.23 to 3.83
5. Aspiration	0 (0%)	5 (6.5%)	6.49%	0.146	0.9 to 11.00
6. Ear pain/hearing loss	1 (3.2%)	8 (10.4%)	7.16%	0.223	−2.06 to 16.39
7. Salty taste	2 (6.4%)	11 (14.3%)	7.83%	0.258	−3.82 to 19.49

**Notes.**

* *p* < 0.05.

CI confidence interval

### Large-volume devices

Large-volume devices can be divided into low-pressure and high-pressure devices. The large-volume low-pressure devices used in this study included syringes (*n* = 201), Neti pots (*n* = 9), and bulbs (*n* = 13), while the large-volume high-pressure devices used were syringes with nasal adapters (*n* = 27) and squeeze bottles (*n* = 50).

#### Symptom scores

The large-volume high-pressure nasal irrigation devices received significantly higher scores in four of the 12 nasal-sympton domains (*p* < 0.05) including overall symptoms (MD 0.62; 95% CI [0.15–1.10), nasal congestion (MD 0.59; 95% CI [0.08–1.11]), pain/headache (MD 1.09; 95% CI [0.30–1.88]), and improve taste/smell (MD 0.89; 95% CI [0.02–1.75]; Table 4).

**Table 4:** Symptom scores for large-volume devices.

**Scale (0–10; higher is better)**	**Large-volume devices**		**Mean difference**	***p*-value**	**95% CI**
	**Low-pressure(*n* = 223)**	**High-pressure(*n* = 77)**			
1. Improve overall symptom	7.84 ± 1.88	8.46 ± 1.60	0.62	0.011^*^	0.15 to 1.10
2. Improve nasal congestion	7.69 ± 1.97	8.28 ± 1.76	0.59	0.024^*^	0.08 to 1.11
3. Decrease runny nose	7.51 ± 2.07	8.06 ± 1.88	0.55	0.050	−0.00 to 1.10
4. Decrease blowing nose	7.60 ± 2.21	7.97 ± 2.17	0.38	0.217	−0.22 to 0.98
5. Decrease viscosity	7.89 ± 2.06	8.32 ± 1.89	0.43	0.119	−0.11 to 0.97
6. Improve sinus pain/headache	6.72 ± 2.75	7.81 ± 2.07	1.09	0.007^*^	0.30 to 1.88
7. Decrease post-nasal drip	7.30 ± 2.23	7.89 ± 2.23	0.59	0.067	−0.04 to 1.23
8. Improve taste and smell	6.90 ± 2.86	7.79 ± 2.60	0.89	0.044^*^	0.02 to 1.75
9. Decrease sneezing	7.46 ± 2.34	7.56 ± 2.31	0.10	0.778	−0.61 to 0.81
10. Decrease cough	7.13 ± 2.48	7.17 ± 2.70	0.04	0.916	−0.77 to 0.86
11. Clear the secretion	7.94 ± 1.96	8.36 ± 1.95	0.41	0.126	−0.12 to 0.95
12. Improve sleep quality	8.01 ± 2.13	8.35 ± 1.88	0.34	0.268	−0.27 to 0.95

**Notes.**

* *p* < 0.05.

CI confidence interval

The multivariate analysis using linear regression was performed to evaluate the impact of sex, age, smoking status, frequency of nasal irrigation, and nasal conditions on the overall symptoms score. However, the result indicated these factors did not reach a statistically significant level of impact on the overall symptoms score (*p* < 0.05).

#### Ease of use, learning curve, and satisfaction

The large-volume high-pressure nasal irrigation devices received the higher scores in the learning curve and patients’ willingness to recommend them to others (MD 0.56; 95% CI [0.05–1.07] and MD 0.67; 95% CI [0.09–1.25], respectively). The mean score for ease of use, learning curve, satisfaction score and recommendation the device to others were more than eight in all items of both groups (scale 0–10 higher is better) (Table 5).

**Table 5:** Ease of use, learning curve, and satisfaction scores of large-volume devices.

**Scale (0–10; higher is better)**	**Large-volume devices**		**Mean difference**	***p*-value**	**95% CI**
	**Low-pressure(*n* = 223)**	**High-pressure(*n* = 77)**			
1. Ease of use	8.40 ± 2.07	8.84 ± 1.74	0.44	0.104	−0.09 to 0.97
2. Learning curve	8.43 ± 2.03	8.98 ± 1.49	0.56	0.031^*^	0.05 to 1.07
3. Satisfaction score	8.56 ± 2.06	8.95 ± 1.85	0.39	0.155	−0.15 to 0.92
4. Would recommend the device to others	8.33 ± 2.30	9 ± 1.75	0.67	0.024^*^	0.09 to 1.25

**Notes.**

* *p* < 0.05.

CI confidence interval

In the subgroup analysis, allergic rhinitis patients rated higher score for ease of use (MD 0.98; 95% CI [0.23–1.73]), learning curve (MD 1.13; 95% CI [0.40–1.86]), satisfaction score (MD 0.86; 95% CI [0.12–1.60]) and recommendation to others (MD 0.87; 95% CI [0.09–1.65]) for large-volume high-pressure nasal irrigation devices. There was no statistical difference between two groups in other nasal diseases.

#### Adverse events

The most common adverse events that accompanied the use of large-volume devices was salty taste (15.2 and 14.3% for low-pressure and high-pressure devices, respectively) and retained fluid in the sinuses (12.9 and 29.9% for low-pressure and high-pressure devices, respectively). A higher proportion of patients using the large-volume high-pressure devices had retained fluid in their sinuses compared to those using large-volume low-pressure devices (*p* < 0.001; Table 6).

**Table 6:** Adverse events of large-volume devices.

	**Large-volume devices**		**Risk difference**	***p*-value**	**95% CI**
	**Low-pressure(*n* = 223)**	**High-pressure(*n* = 77)**			
1. Pain/discomfort	16 (7.2%)	1 (1.2%)	−5.88%	0.055	−10.10 to −1.65
2. Retained fluid in sinuses	29 (13.0%)	23 (29.9%)	16.87%	<0.001^*^	5.73 to 28.00
3. Epistaxis	6 (2.7%)	1 (1.3%)	−1.39%	0.486	−4.69 to 1.91
4. Headache	14 (6.3%)	1 (1.3%)	−4.98%	0.084	−9.05to -0.91
5. Aspiration	25 (11.2%)	5 (6.5%)	−4.71%	0.234	−11.60 to 2.17
6. Ear pain/hearing loss	23 (10.3%)	8 (10.4%)	0.08%	0.985	−7.82 to 7.97
7. Salty taste	34 (15.2%)	11 (14.3%)	−0.96%	0.839	−10.09 to 8.17

**Notes.**

* *p* < 0.05.

CI confidence interval

### Physician survey

The 90 questionnaires were collected from the physicians at universities in three different areas of Thailand: 23 from Khon Kaen University, 32 from Chiangmai University, and 35 from Prince of Songkhla University according to the number of Otolaryngology residents and staff at each centre. All of them were agreed to provide their experience in this study.

Most physicians prescribed the syringe, syringe with nasal adapter, squeeze bottle, and spray (91.1, 41.1, 12.2%, and 11.1%, respectively). They recommended nasal irrigation in various kinds of nasal diseases including chronic sinusitis, postnasal operation, acute sinusitis, and allergic rhinitis (87.8, 84.4, 73.3, and 64.4%, respectively).

## Discussion

As part of a national review on the efficacy of nasal saline irrigation, we performed a survey to determine the effectiveness and safety of various nasal irrigation devices from the perspective of patients and physicians according to their previous experience.

This program made us realise the limited availability of evidence regarding nasal irrigation in current practice. There has yet been no consensus as to which the best irrigation device is, as there has not yet been enough randomised controlled trials or controlled clinical trials directly comparing each device.

Campos, Heppt & Weber (2013) performed an *in-vitro* comparison of the irrigation devices that were available on the market by testing them on a nasal cavity model. They found that the greater the volume and pressure, the higher chance the saline would reach entire nasal cavity.

Wormald et al. (2004) compared nasal spray, nebulization, and nasal douching in 21 subjects (nine patients with chronic sinusitis after functional endoscopic sinus surgery and three healthy controls). They found that douching was significantly more effective in penetrating the maxillary sinus (*p* = 0.036) and frontal recess (*p* = 0.003). The sphenoid and frontal sinuses were poorly irrigated by all three techniques.

Salib et al. performed a prospective randomised single-blinded clinical trial comparing the efficacy and tolerability of nasal douching using low-volume high-pressure and high-volume low-pressure devices following functional endoscopic sinus surgery in 31 patients. They found a significant improvement in nasal findings scores at 2 and 4 weeks in the high-volume low-pressure group. However, the effect was diminished at 12 weeks postoperatively (Salib et al., 2013). In a recent Cochrane’s Review for allergic rhinitis, subgroup analysis was performed for volume and tonicity, but the results were inconclusive due to the heterogeneity (Head et al., 2018).

We found that the high-pressure large-volume nasal irrigation devices received significantly higher scores than high-pressure low-volume device in seven of 12 domains (*p* < 0.05). The high-pressure large-volume nasal irrigation devices were also recommended by the patients (*p* < 0.05). There was no statistically significant difference in adverse events among device subgroups.

We also found the large-volume high-pressure nasal irrigation devices received significantly higher scores than the large-volume low-pressure devices in four of 12 domains (*p* < 0.05). The large-volume high-pressure nasal irrigation devices received higher scores in ease of learning and patients’ willingness to recommend them to others (*p* < 0.05). However, a higher proportion of the patients who used large-volume high-pressure devices had retained fluid in the sinuses compared to those who used large-volume low-pressure devices (*p* < 0.001).

As we expected based on the *in-vitro* data, the large-volume high-pressure nasal irrigation devices received the best scores compared to other types of device, as the volume and pressure of the saline are able to effectively clear the mucus in the nasal and sinus cavities. However, the patients will have to accept that they may experience retained fluid in the sinuses, which is a minor adverse effect when using these devices.

Most patients recommended high-pressure large-volume devices to others for nasal irrigation (*p* < 0.05). The patient’s recommendation was aggregate outcome based on whether the person was happy doing the procedure, and whether there was symptomatic relief. The symptomatic relief may be depending on other variables such as sex, age, smoking status, frequency of nasal irrigation, and nasal conditions. We have performed the multivariate analysis and found no significant impact of these factors on the overall symptoms score.

There was an argument that the syringe without nasal adapter in an experienced hand can hold the syringe in the exact position that can avoid a loss of pressure from solution leakage and should be categorised as a high-pressure device. In our unreported data comparing syringe alone versus the syringe with adapter, patients reported the preference of syringe with adapter over syringe alone as it can snugly fit in the nostril and no solution leakage from their nose.

The participants in this study were recruited from the university hospital and their peer otolaryngologist specialists. The sample from the university hospital and specialist clinics usually had more complex or serious illnesses compared with community hospital or general practice clinic. The future multicentre trial should address this problem by including the participants in a general practice setting.

This study was a multi-centre survey from all major regions of Thailand comparing common nasal irrigation devices used in the country. Although these results can likely apply in other Asian countries, their implications beyond the study population may be limited.

## Conclusions

This survey supports the regular use of nasal irrigation, particularly with large-volume high-pressure devices, as an effective treatment for nasal disease. It may be effective at clearing nasal secretion, improve nasal congestion, decrease post-nasal drip, improve sinus pain or headache, improve taste and smell, and improve sleep quality. The allergic rhinitis patients are likely to get more benefit from large-volume high-pressure devices as indicated by a better ease of use, learning curve, and satisfaction score.

Large-volume high-pressure devices could be used as an add-on therapy to standard treatment for the nasal disease with good compliance and minimal side effects.

##  Supplemental Information

10.7717/peerj.7000/supp-1 Supplemental Information 1 Raw data of Thai national survey of nasal irrigation devices

10.7717/peerj.7000/supp-2 Supplemental Information 2 Codebook

10.7717/peerj.7000/supp-3 Supplemental Information 3 Patient questionnaire (Thai)

10.7717/peerj.7000/supp-4 Supplemental Information 4 Physician questionnaire (Thai)

## Additional Information and Declarations

### Data Availability

The following information was supplied regarding data availability: The raw data are available in Supplementary File 1.
