# Supplementary material for: Effectiveness of nasal irrigation devices: a Thai multicentre survey
Source: PeerJ. 2019 May 27;7:e7000. doi: 10.7717/peerj.7000 (PMC6542345; doi:10.7717/peerj.7000)
Supplement: Supplemental Information 2 [file peerj-07-7000-s002.pdf]

Codebook

Variables	Description	Value label
ID	ID	
Site	Major site and subsite of recruitment	
a1gender	Gender	1 = male, 2 = female
a2age	Age	
a3smoke	Smoking	0 = no, 1 = yes
a4device	Devices used	1 = syringe 2 = syringe with adapter 3 = squeeze bottle 4 = Neti pot 5 = bulb 6 = Spray 7 = Drops
b1disease1	Nasal diseases	1. Allergic rhinitis 2. Acute rhinosinusitis 3. Chronic rhinosinusitis 4. Post-operative 5. Common cold. 6. Others….
b1disease2	Nasal diseases	1. Allergic rhinitis 2. Acute rhinosinusitis 3. Chronic rhinosinusitis 4. Post-operative 5. Common cold. 6. Others….
b1disease3	Nasal diseases	1. Allergic rhinitis 2. Acute rhinosinusitis 3. Chronic rhinosinusitis 4. Post-operative 5. Common cold. 6. Others….
b2timeMonth	Have been using this device for	
b3frequencyDay	Frequency of use	
b4irrigate	You use the device	1. regularly, 2. When have nasal symptoms, 3. Other
b5teach1	Who teaches you to use the device	1. Physician 2. Nurse 3. Pharmacist 4. Parent 5. Self-learning
b5teach2	Who teaches you to use the device	1. Physician 2. Nurse 3. Pharmacist 4. Parent 5. Self-learning
b5teach3	Who teaches you to use the device	1. Physician 2. Nurse 3. Pharmacist 4. Parent 5. Self-learning
b60	The problem of this device	0. No 1. Nasal pain 2. Solution retention 3. Epistaxis 4. Headache 5. Aspiration 6. Hearing loss 7. Salty taste 8. Other
b61	The problem of this device	0. No 1. Nasal pain 2. Solution retention 3. Epistaxis 4. Headache 5. Aspiration 6. Hearing loss 7. Salty taste 8. Other

b62	The problem of this device	0. No 1. Nasal pain 2. Solution retention 3. Epistaxis 4. Headache 5. Aspiration 6. Hearing loss 7. Salty taste 8. Other
b63	The problem of this device	0. No 1. Nasal pain 2. Solution retention 3. Epistaxis 4. Headache 5. Aspiration 6. Hearing loss 7. Salty taste 8. Other
b64	The problem of this device	0. No 1. Nasal pain 2. Solution retention 3. Epistaxis 4. Headache 5. Aspiration 6. Hearing loss 7. Salty taste 8. Other
b65	The problem of this device	0. No 1. Nasal pain 2. Solution retention 3. Epistaxis 4. Headache 5. Aspiration 6. Hearing loss 7. Salty taste 8. Other
b66	The problem of this device	0. No 1. Nasal pain 2. Solution retention 3. Epistaxis 4. Headache 5. Aspiration 6. Hearing loss 7. Salty taste 8. Other
b67	The problem of this device	0. No 1. Nasal pain 2. Solution retention 3. Epistaxis 4. Headache 5. Aspiration 6. Hearing loss 7. Salty taste 8. Other
b68	The problem of this device	0. No 1. Nasal pain 2. Solution retention 3. Epistaxis 4. Headache 5. Aspiration 6. Hearing loss 7. Salty taste 8. Other
b69	The problem of this device	0. No 1. Nasal pain 2. Solution retention 3. Epistaxis 4. Headache 5. Aspiration 6. Hearing loss 7. Salty taste 8. Other
b7posture	Position	1. Flex neck 2. Extend neck 3. Other
b8breath1	Do you breathe when performing irrigation	1. Nasal breath 2. Hold the breath 3. Oral breathe 4. Other
b8breath2	Do you breathe when performing irrigation	1. Nasal breath 2. Hold the breath 3. Oral breathe 4. Other
b9brand	Name of the device	
b10brokeMonth	The device broke in	
b11clean1	You clean the device using	1. Soap 2. Saline 3. Warm water 4. Boil 5. Other
b11clean2	You clean the device using	1. Soap 2. Saline 3. Warm water 4. Boil 5. Other
b12get1	You got this device from	1. Hospital 2. Pharmacist 3. Clinic
b12get2	You got this device from	1. Hospital 2. Pharmacist 3. Clinic

b12get3	You got this device from	1. Hospital 2. Pharmacist 3. Clinic
c1	Improve overall symptom	
c2symptom	No nasal congestion before	0. No 1. Yes
c2	Improve nasal congestion	
c3symptom	No runny nose before	0. No 1. Yes
c3	Decrease runny nose	
c4symptom	No need to blowing nose before	0. No 1. Yes
c4	Decrease blowing nose	
c5symptom	No nasal section before	0. No 1. Yes
c5	Decrease viscosity	
c6symptom	No pain before	0. No 1. Yes
c6	Improve sinus pain/headache	
c7symptom	No post-nasal drip before	0. No 1. Yes
c7	Decrease post-nasal drip	
c8symptom	No taste/smell disturbed before	0. No 1. Yes
c8	Improve taste and smell	
c9symptom	No sneezing before	0. No 1. Yes
c9	Decrease sneezing	
c10symptom	No cough before	0. No 1. Yes
c10	Decrease cough	
c11symptom	No nasal section before	0. No 1. Yes
c11	Clear the secretion	
c12symptom	No sleep abnormality before	0. No 1. Yes
c12	Improve sleep quality	
c13	Easy to use	
c14	Simple	
c15	Fast learning	
c16	Can remember the instruction	
c17	Satisfaction of this device	
c18	Would recommend the device to others	
