# Supplementary material for: Effectiveness of nasal irrigation devices: a Thai multicentre survey
Source: PeerJ. 2019 May 27;7:e7000. doi: 10.7717/peerj.7000 (PMC6542345; doi:10.7717/peerj.7000)
Supplement: Supplemental Information 3 [file peerj-07-7000-s003.pdf]

แบบสอบถามความพึงพอใจของผู้ป่วยต่อการล้างจมูก

****หากท่านเคยตอบแบบสอบถามนี้แล้ว ไม่ต้องทำซ้ำ กรุณาส่งคืน****

<u>ส่วนที่ 1 ข้อมูลทั่วไป</u>

1. เพศ ❑ 1.ชาย ❑ 2.หญิง
2. อายุ ☐☐ ปี
3. สูบบุหรี่ ❑ 0.ไม่สูบ ❑ 1.สูบ

อุปกรณ์ล้างจมูกที่เคยใช้ (เลือกได้มากกว่า 1 อย่าง)

❑ 1. หลอดฉีดยา

กรุณาตอบคำถามที่หน้า 2

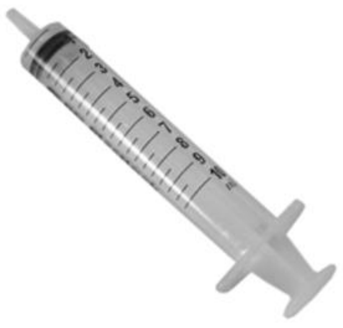

❑ 2. หลอดฉีดยามีจุก

กรุณาตอบคำถามที่หน้า 4

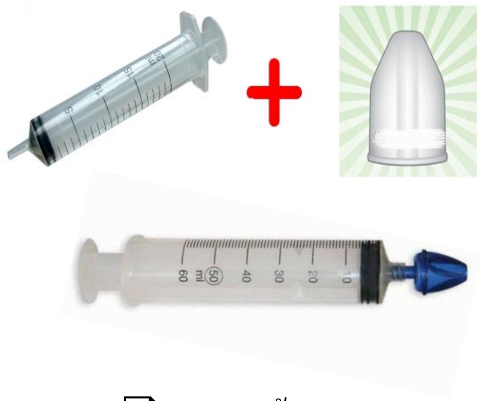

❑ 3. ขวดล้างจมูกแบบบีบ

กรุณาตอบคำถามที่หน้า 6

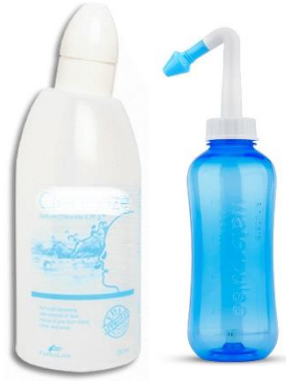

❑ 4. ขวดล้างจมูกแบบเท บีบไม่ได้

กรุณาตอบคำถามที่หน้า 8

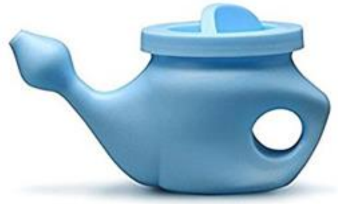

❑ 5. ลูกยางล้างจมูก

กรุณาตอบคำถามที่หน้า 10

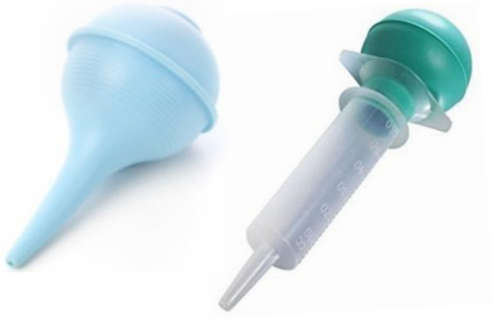

❑ 6. สเปรย์ล้างจมูก

กรุณาตอบคำถามที่หน้า 12

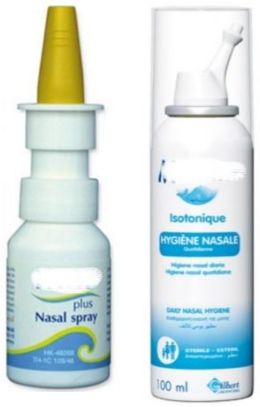

❑ 7. ที่หยดล้างจมูก

กรุณาตอบคำถามที่หน้า 14

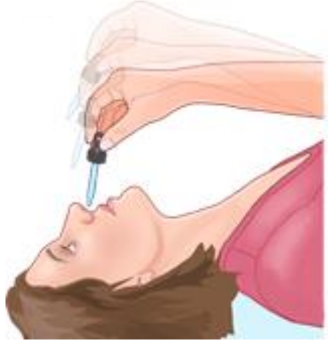

แบบสอบถามที่ 1 หลอดฉีดยา

1. คุณเคยใช้อุปกรณ์นี้ในการรักษาโรคใด

❑ 1. จมูกอักเสบจากภูมิแพ้ ❑ 2. ไซนัสอักเสบเฉียบพลัน ❑ 3. ไซนัสอักเสบเรื้อรัง

❑ 4. หลังผ่าตัด ❑ 5. ไข้หวัด ❑ 6. อื่น ๆ โปรดระบุ.......................................

2. คุณใช้อุปกรณ์นี้มานาน ☐☐ ปี ☐☐ เดือน

3. ความถี่ในการใช้ล้างจมูกโดยประมาณ ☐☐ ครั้ง/วัน หรือ ☐☐ ครั้ง/สัปดาห์

4. คุณล้างจมูกบ่อยแค่ไหน (โดยประมาณ) ❑ 1. ล้างทุกวัน ❑ 2. ล้างเมื่อมีอาการ ❑3.อื่น ๆ ....

5. ใครเป็นผู้สอนคุณล้างจมูก ❑ 1. แพทย์ ❑ 2. พยาบาล ❑ 3. เภสัชกร ❑ 4. ผู้ปกครอง ❑ 5. เรียนรู้เอง

6. ปัญหาของการใช้อุปกรณ์นี้ (ตอบได้มากกว่า 1 ข้อ)

❑ 0. ไม่มี ❑ 1. เจ็บจมูก ❑ 2. น้ำเกลือค้างในจมูก ❑ 3. เลือดกำเดาไหล ❑ 4. ปวดศีรษะ

❑ 5.สำลัก ❑ 6. หูอื้อ ❑ 7. เค็มคอ ❑ 8. อื่น ๆ โปรดระบุ……………………..

7. ท่าที่ใช้ในการล้างจมูก ❑ 1. ก้มหน้า ❑ 2. เงยหน้า ❑ 3. อื่น ๆ…………………………………

8. ระหว่างล้างน้ำเกลือ (น้ำเกลือเข้าจมูก) คุณหายใจหรือไม่

❑ 1. หายใจทางจมูก ❑ 2. กลั้นหายใจ ❑ 3. หายใจทางปาก ❑ 4. อื่น ๆ ....

9. ยี่ห้ออุปกรณ์ที่คุณเคยใช้ ………………………………………

10. ใช้อุปกรณ์นี้ไปได้นานเท่าไร อุปกรณ์จึงเสีย ☐☐ ปี ☐☐ เดือน

11. คุณทำความสะอาดอุปกรณ์นี้อย่างไร ❑ ล้างน้ำสบู่ ❑ ล้างน้ำเกลือ ❑ ล้างน้ำอุ่น ❑ ต้ม ❑ อื่น ๆ…………..

12. คุณได้อุปกรณ์นี้มาจากที่ใด ❑ โรงพยาบาล ❑ ร้านขายยา ❑ คลินิก

คำชี้แจง

วงกลม ◯ ล้อมรอบตัวเลข ในช่องด้านขวามือที่ท่านพิจารณาว่าเป็นข้อความที่ตรงกับความรู้สึกในขณะนี้

**ความมีประโยชน์ทางคลินิกของอุปกรณ์ล้างจมูก**	**ไม่เห็นด้วยที่สุด** **0** — **เห็นด้วยที่สุด** **10**
1.คุณรู้สึกว่าอุปกรณ์นี้ช่วยให้อาการทางจมูกโดยรวมของคุณลดลง	0 1 2 3 4 5 6 7 8 9 10
2.คุณรู้สึกว่าอุปกรณ์นี้ช่วยให้อาการคัดจมูกของคุณลดลง	0 1 2 3 4 5 6 7 8 9 10 ❑ ไม่มีอาการคัดจมูกมาก่อน
3.คุณรู้สึกว่าอุปกรณ์นี้ช่วยให้อาการน้ำมูกไหลของคุณลดลง	0 1 2 3 4 5 6 7 8 9 10 ❑ ไม่มีอาการน้ำมูกไหลมาก่อน
4.คุณรู้สึกว่าอุปกรณ์นี้ช่วยให้คุณสั่งน้ำมูกลดลง	0 1 2 3 4 5 6 7 8 9 10 ❑ ไม่ต้องสั่งน้ำมูกมาก่อน
5.คุณรู้สึกว่าอุปกรณ์นี้ช่วยทำให้น้ำมูกข้นน้อยลง	0 1 2 3 4 5 6 7 8 9 10 ❑ ไม่มีน้ำมูกข้นมาก่อน

6.คุณรู้สึกว่าอุปกรณ์นี้ช่วยให้อาการปวดบริเวณใบหน้าน้อยลง	0 1 2 3 4 5 6 7 8 9 10	❑ ไม่มีปวดใบหน้ามาก่อน
7คุณรู้สึกว่าอุปกรณ์นี้ช่วยให้น้ำมูกไหลลงคอลดลง	0 1 2 3 4 5 6 7 8 9 10	❑ ไม่มีน้ำมูกไหลลงคอมาก่อน
8.คุณรู้สึกว่าอุปกรณ์นี้ช่วยให้การได้กลิ่นและการรับรสดีขึ้น	0 1 2 3 4 5 6 7 8 9 10	❑ ไม่มีการได้กลิ่นและรับรสที่ผิดปกติมาก่อน
9.คุณรู้สึกว่าอุปกรณ์นี้ช่วยให้อาการจามลดลง	0 1 2 3 4 5 6 7 8 9 10	❑ ไม่มีอาการจามมาก่อน
10.คุณรู้สึกว่าอุปกรณ์นี้ช่วยให้อาการไอลดลง	0 1 2 3 4 5 6 7 8 9 10	❑ ไม่มีอาการไอมาก่อน
11.คุณรู้สึกว่าอุปกรณ์นี้เข้าไปชะล้างน้ำมูกได้ทั่วถึง	0 1 2 3 4 5 6 7 8 9 10	❑ ไม่มีน้ำมูกมาก่อน
12.คุณรู้สึกว่าอุปกรณ์นี้ช่วยให้การนอนโดยรวมของคุณดีขึ้น	0 1 2 3 4 5 6 7 8 9 10	❑ ไม่มีการนอนที่ผิดปกติมาก่อน
**ความสะดวกในการใช้งาน**		
13.คุณรู้สึกว่าอุปกรณ์นี้ใช้งานง่าย	0 1 2 3 4 5 6 7 8 9 10	
14.คุณสามารถใช้งานอุปกรณ์ล้างจมูกนี้ได้โดยที่ไม่ต้องมีคำแนะนำวิธีใช้เขียนกำกับไว้	0 1 2 3 4 5 6 7 8 9 10	
**การเรียนรู้วิธีใช้**		
15.คุณสามารถเรียนรู้วิธีการใช้อุปกรณ์ล้างจมูกนี้ได้อย่างรวดเร็ว	0 1 2 3 4 5 6 7 8 9 10	
16.คุณสามารถจำวิธีการใช้อุปกรณ์ล้างจมูกได้	0 1 2 3 4 5 6 7 8 9 10	
**ความพึงพอใจ**		
17.คุณมีความพึงพอใจในการใช้อุปกรณ์ล้างจมูกชนิดนี้	0 1 2 3 4 5 6 7 8 9 10	
18.คุณจะแนะนำอุปกรณ์ล้างจมูกที่คุณใช้อยู่นี้ให้แก่เพื่อนของคุณ	0 1 2 3 4 5 6 7 8 9 10	

แบบสอบถามที่ 2 หลอดฉีดยามีจุก

1. คุณเคยใช้อุปกรณ์นี้ในการรักษาโรคใด

❑ 1. จมูกอักเสบจากภูมิแพ้ ❑ 2. ไซนัสอักเสบเฉียบพลัน ❑ 3. ไซนัสอักเสบเรื้อรัง

❑ 4. หลังผ่าตัด ❑ 5. ไข้หวัด ❑ 6. อื่น ๆ โปรดระบุ.......................................

2. คุณใช้อุปกรณ์นี้มานาน ☐☐ ปี ☐☐ เดือน

3. ความถี่ในการใช้ล้างจมูกโดยประมาณ ☐☐ ครั้ง/วัน หรือ ☐☐ ครั้ง/สัปดาห์

4. คุณล้างจมูกบ่อยแค่ไหน (โดยประมาณ) ❑ 1. ล้างทุกวัน ❑ 2. ล้างเมื่อมีอาการ ❑3.อื่น ๆ ....

5. ใครเป็นผู้สอนคุณล้างจมูก ❑ 1. แพทย์ ❑ 2. พยาบาล ❑ 3. เภสัชกร ❑ 4. ผู้ปกครอง ❑ 5. เรียนรู้เอง

6. ปัญหาของการใช้อุปกรณ์นี้ (ตอบได้มากกว่า 1 ข้อ)

❑ 0. ไม่มี ❑ 1. เจ็บจมูก ❑ 2. น้ำเกลือค้างในจมูก ❑ 3. เลือดกำเดาไหล ❑ 4. ปวดศีรษะ

❑ 5.สำลัก ❑ 6. หูอื้อ ❑ 7. เค็มคอ ❑ 8. จุกขาด ❑ อื่น ๆ โปรดระบุ………………

7. ท่าที่ใช้ในการล้างจมูก ❑ 1. ก้มหน้า ❑ 2. เงยหน้า ❑ 3. อื่น ๆ…………………………………

8. ระหว่างล้างน้ำเกลือ (น้ำเกลือเข้าจมูก) คุณหายใจหรือไม่

❑ 1. หายใจทางจมูก ❑ 2. กลั้นหายใจ ❑ 3. หายใจทางปาก ❑ 4. อื่น ๆ ....

9. ยี่ห้ออุปกรณ์ที่คุณเคยใช้ ………………………………………

10. ใช้อุปกรณ์นี้ไปได้นานเท่าไร อุปกรณ์จึงเสีย ☐☐ ปี ☐☐ เดือน

11. คุณทำความสะอาดอุปกรณ์นี้อย่างไร ❑ ล้างน้ำสบู่ ❑ ล้างน้ำเกลือ ❑ ล้างน้ำอุ่น ❑ ต้ม ❑ อื่น ๆ…………..

12. คุณได้อุปกรณ์นี้มาจากที่ใด ❑ โรงพยาบาล ❑ ร้านขายยา ❑ คลินิก

คำชี้แจง

วงกลม ○ ล้อมรอบตัวเลข ในช่องด้านขวามือที่ท่านพิจารณาว่าเป็นข้อความที่ตรงกับความรู้สึกในขณะนี้

**ความมีประโยชน์ทางคลินิกของอุปกรณ์ล้างจมูก**	**ไม่เห็นด้วยที่สุด** 0 — **เห็นด้วยที่สุด** 10	
1.คุณรู้สึกว่าอุปกรณ์นี้ช่วยให้อาการทางจมูกโดยรวมของคุณลดลง	0 1 2 3 4 5 6 7 8 9 10	
2.คุณรู้สึกว่าอุปกรณ์นี้ช่วยให้อาการคัดจมูกของคุณลดลง	0 1 2 3 4 5 6 7 8 9 10	❑ ไม่มีอาการคัดจมูกมาก่อน
3.คุณรู้สึกว่าอุปกรณ์นี้ช่วยให้อาการน้ำมูกไหลของคุณลดลง	0 1 2 3 4 5 6 7 8 9 10	❑ ไม่มีอาการน้ำมูกไหลมาก่อน
4.คุณรู้สึกว่าอุปกรณ์นี้ช่วยให้คุณสั่งน้ำมูกลดลง	0 1 2 3 4 5 6 7 8 9 10	❑ ไม่ต้องสั่งน้ำมูกมาก่อน
5.คุณรู้สึกว่าอุปกรณ์นี้ช่วยทำให้น้ำมูกข้นน้อยลง	0 1 2 3 4 5 6 7 8 9 10	❑ ไม่มีน้ำมูกข้นมาก่อน

6.คุณรู้สึกว่าอุปกรณ์นี้ช่วยให้อาการปวดบริเวณใบหน้าน้อยลง	0 1 2 3 4 5 6 7 8 9 10	❑ ไม่มีปวดใบหน้ามาก่อน
7คุณรู้สึกว่าอุปกรณ์นี้ช่วยให้น้ำมูกไหลลงคอลดลง	0 1 2 3 4 5 6 7 8 9 10	❑ ไม่มีน้ำมูกไหลลงคอมาก่อน
8.คุณรู้สึกว่าอุปกรณ์นี้ช่วยให้การได้กลิ่นและการรับรสดีขึ้น	0 1 2 3 4 5 6 7 8 9 10	❑ ไม่มีการได้กลิ่นและรับรสที่ผิดปกติมาก่อน
9.คุณรู้สึกว่าอุปกรณ์นี้ช่วยให้อาการจามลดลง	0 1 2 3 4 5 6 7 8 9 10	❑ ไม่มีอาการจามมาก่อน
10.คุณรู้สึกว่าอุปกรณ์นี้ช่วยให้อาการไอลดลง	0 1 2 3 4 5 6 7 8 9 10	❑ ไม่มีอาการไอมาก่อน
11.คุณรู้สึกว่าอุปกรณ์นี้เข้าไปชะล้างน้ำมูกได้ทั่วถึง	0 1 2 3 4 5 6 7 8 9 10	❑ ไม่มีน้ำมูกมาก่อน
12.คุณรู้สึกว่าอุปกรณ์นี้ช่วยให้การนอนโดยรวมของคุณดีขึ้น	0 1 2 3 4 5 6 7 8 9 10	❑ ไม่มีการนอนที่ผิดปกติมาก่อน
**ความสะดวกในการใช้งาน**		
13.คุณรู้สึกว่าอุปกรณ์นี้ใช้งานง่าย	0 1 2 3 4 5 6 7 8 9 10	
14.คุณสามารถใช้งานอุปกรณ์ล้างจมูกนี้ได้โดยที่ไม่ต้องมีคำแนะนำวิธีใช้เขียนกำกับไว้	0 1 2 3 4 5 6 7 8 9 10	
**การเรียนรู้วิธีใช้**		
15.คุณสามารถเรียนรู้วิธีการใช้อุปกรณ์ล้างจมูกนี้ได้อย่างรวดเร็ว	0 1 2 3 4 5 6 7 8 9 10	
16.คุณสามารถจำวิธีการใช้อุปกรณ์ล้างจมูกได้	0 1 2 3 4 5 6 7 8 9 10	
**ความพึงพอใจ**		
17.คุณมีความพึงพอใจในการใช้อุปกรณ์ล้างจมูกชนิดนี้	0 1 2 3 4 5 6 7 8 9 10	
18.คุณจะแนะนำอุปกรณ์ล้างจมูกที่คุณใช้อยู่นี้ให้แก่เพื่อนของคุณ	0 1 2 3 4 5 6 7 8 9 10	

แบบสอบถามที่ 3 ขวดล้างจมูกแบบบีบ

1. คุณเคยใช้อุปกรณ์นี้ในการรักษาโรคใด

❑ 1. จมูกอักเสบจากภูมิแพ้ ❑ 2. ไซนัสอักเสบเฉียบพลัน ❑ 3. ไซนัสอักเสบเรื้อรัง

❑ 4. หลังผ่าตัด ❑ 5. ไข้หวัด ❑ 6. อื่น ๆ โปรดระบุ.......................................

2. คุณใช้อุปกรณ์นี้มานาน ☐☐ ปี ☐☐ เดือน

3. ความถี่ในการใช้ล้างจมูกโดยประมาณ ☐☐ ครั้ง/วัน หรือ ☐☐ ครั้ง/สัปดาห์

4. คุณล้างจมูกบ่อยแค่ไหน (โดยประมาณ) ❑ 1. ล้างทุกวัน ❑ 2. ล้างเมื่อมีอาการ ❑3.อื่น ๆ ....

5. ใครเป็นผู้สอนคุณล้างจมูก ❑ 1. แพทย์ ❑ 2. พยาบาล ❑ 3. เภสัชกร ❑ 4. ผู้ปกครอง ❑ 5. เรียนรู้เอง

6. ปัญหาของการใช้อุปกรณ์นี้ (ตอบได้มากกว่า 1 ข้อ)

❑ 0. ไม่มี ❑ 1. เจ็บจมูก ❑ 2. น้ำเกลือค้างในจมูก ❑ 3. เลือดกำเดาไหล ❑ 4. ปวดศีรษะ

❑ 5.สำลัก ❑ 6. หูอื้อ ❑ 7. เค็มคอ ❑ 8. ขวดแตก ❑9.บีบยาก

❑ อื่น ๆ โปรดระบุ……………………..

7. ท่าที่ใช้ในการล้างจมูก ❑ 1. ก้มหน้า ❑ 2. เงยหน้า ❑ 3. อื่น ๆ…………………………………

8. ระหว่างล้างน้ำเกลือ (น้ำเกลือเข้าจมูก) คุณหายใจหรือไม่

❑ 1. หายใจทางจมูก ❑ 2. กลั้นหายใจ ❑ 3. หายใจทางปาก ❑ 4. อื่น ๆ ....

9. ยี่ห้ออุปกรณ์ที่คุณเคยใช้ ....

10. ใช้อุปกรณ์นี้ไปได้นานเท่าไร อุปกรณ์จึงเสีย ☐☐ ปี ☐☐ เดือน

11. คุณทำความสะอาดอุปกรณ์นี้อย่างไร ❑ ล้างน้ำสบู่ ❑ ล้างน้ำเกลือ ❑ ล้างน้ำอุ่น ❑ ต้ม ❑ อื่น ๆ…………..

12. คุณได้อุปกรณ์นี้มาจากที่ใด ❑ โรงพยาบาล ❑ ร้านขายยา ❑ คลินิก

คำชี้แจง

วงกลม ◯ ล้อมรอบตัวเลข ในช่องด้านขวามือที่ท่านพิจารณาว่าเป็นข้อความที่ตรงกับความรู้สึกในขณะนี้

**ความมีประโยชน์ทางคลินิกของอุปกรณ์ล้างจมูก**	**ไม่เห็นด้วยที่สุด** **0** — **เห็นด้วยที่สุด** **10**	
1.คุณรู้สึกว่าอุปกรณ์นี้ช่วยให้อาการทางจมูกโดยรวมของคุณลดลง	0 1 2 3 4 5 6 7 8 9 10	
2.คุณรู้สึกว่าอุปกรณ์นี้ช่วยให้อาการคัดจมูกของคุณลดลง	0 1 2 3 4 5 6 7 8 9 10	❑ ไม่มีอาการคัดจมูกมาก่อน
3.คุณรู้สึกว่าอุปกรณ์นี้ช่วยให้อาการน้ำมูกไหลของคุณลดลง	0 1 2 3 4 5 6 7 8 9 10	❑ ไม่มีอาการน้ำมูกไหลมาก่อน
4.คุณรู้สึกว่าอุปกรณ์นี้ช่วยให้คุณสั่งน้ำมูกลดลง	0 1 2 3 4 5 6 7 8 9 10	❑ ไม่ต้องสั่งน้ำมูกมาก่อน
5.คุณรู้สึกว่าอุปกรณ์นี้ช่วยทำให้น้ำมูกข้นน้อยลง	0 1 2 3 4 5 6 7 8 9 10	❑ ไม่มีน้ำมูกข้นมาก่อน

6.คุณรู้สึกว่าอุปกรณ์นี้ช่วยให้อาการปวดบริเวณใบหน้าน้อยลง	0 1 2 3 4 5 6 7 8 9 10	❑ ไม่มีปวดใบหน้ามาก่อน
7คุณรู้สึกว่าอุปกรณ์นี้ช่วยให้น้ำมูกไหลลงคอลดลง	0 1 2 3 4 5 6 7 8 9 10	❑ ไม่มีน้ำมูกไหลลงคอมาก่อน
8.คุณรู้สึกว่าอุปกรณ์นี้ช่วยให้การได้กลิ่นและการรับรสดีขึ้น	0 1 2 3 4 5 6 7 8 9 10	❑ ไม่มีการได้กลิ่นและรับรสที่ผิดปกติมาก่อน
9.คุณรู้สึกว่าอุปกรณ์นี้ช่วยให้อาการจามลดลง	0 1 2 3 4 5 6 7 8 9 10	❑ ไม่มีอาการจามมาก่อน
10.คุณรู้สึกว่าอุปกรณ์นี้ช่วยให้อาการไอลดลง	0 1 2 3 4 5 6 7 8 9 10	❑ ไม่มีอาการไอมาก่อน
11.คุณรู้สึกว่าอุปกรณ์นี้เข้าไปชะล้างน้ำมูกได้ทั่วถึง	0 1 2 3 4 5 6 7 8 9 10	❑ ไม่มีน้ำมูกมาก่อน
12.คุณรู้สึกว่าอุปกรณ์นี้ช่วยให้การนอนโดยรวมของคุณดีขึ้น	0 1 2 3 4 5 6 7 8 9 10	❑ ไม่มีการนอนที่ผิดปกติมาก่อน
**ความสะดวกในการใช้งาน**		
13.คุณรู้สึกว่าอุปกรณ์นี้ใช้งานง่าย	0 1 2 3 4 5 6 7 8 9 10	
14.คุณสามารถใช้งานอุปกรณ์ล้างจมูกนี้ได้โดยที่ไม่ต้องมีคำแนะนำวิธีใช้เขียนกำกับไว้	0 1 2 3 4 5 6 7 8 9 10	
**การเรียนรู้วิธีใช้**		
15.คุณสามารถเรียนรู้วิธีการใช้อุปกรณ์ล้างจมูกนี้ได้อย่างรวดเร็ว	0 1 2 3 4 5 6 7 8 9 10	
16.คุณสามารถจำวิธีการใช้อุปกรณ์ล้างจมูกได้	0 1 2 3 4 5 6 7 8 9 10	
**ความพึงพอใจ**		
17.คุณมีความพึงพอใจในการใช้อุปกรณ์ล้างจมูกชนิดนี้	0 1 2 3 4 5 6 7 8 9 10	
18.คุณจะแนะนำอุปกรณ์ล้างจมูกที่คุณใช้อยู่นี้ให้แก่เพื่อนของคุณ	0 1 2 3 4 5 6 7 8 9 10	

แบบสอบถามที่ 4 ขวดล้างจมูกแบบเท บีบไม่ได้

1. คุณเคยใช้อุปกรณ์นี้ในการรักษาโรคใด

❑ 1. จมูกอักเสบจากภูมิแพ้ ❑ 2. ไซนัสอักเสบเฉียบพลัน ❑ 3. ไซนัสอักเสบเรื้อรัง

❑ 4. หลังผ่าตัด ❑ 5. ไข้หวัด ❑ 6. อื่น ๆ โปรดระบุ..

2. คุณใช้อุปกรณ์นี้มานาน ☐☐ ปี ☐☐ เดือน

3. ความถี่ในการใช้ล้างจมูกโดยประมาณ ☐☐ ครั้ง/วัน หรือ ☐☐ ครั้ง/สัปดาห์

4. คุณล้างจมูกบ่อยแค่ไหน (โดยประมาณ) ❑ 1. ล้างทุกวัน ❑ 2. ล้างเมื่อมีอาการ ❑3.อื่น ๆ ....

5. ใครเป็นผู้สอนคุณล้างจมูก ❑ 1. แพทย์ ❑ 2. พยาบาล ❑ 3. เภสัชกร ❑ 4. ผู้ปกครอง ❑ 5. เรียนรู้เอง

6. ปัญหาของการใช้อุปกรณ์นี้ (ตอบได้มากกว่า 1 ข้อ)

❑ 0. ไม่มี ❑ 1. เจ็บจมูก ❑ 2. น้ำเกลือค้างในจมูก ❑ 3. เลือดกำเดาไหล ❑ 4. ปวดศีรษะ

❑ 5.สำลัก ❑ 6. หูอื้อ ❑ 7. เค็มคอ ❑ 8. อื่น ๆ โปรดระบุ……………………..

7. ท่าที่ใช้ในการล้างจมูก ❑ 1. ก้มหน้า ❑ 2. เงยหน้า ❑ 3. อื่น ๆ…………………………………

8. ระหว่างล้างน้ำเกลือ (น้ำเกลือเข้าจมูก) คุณหายใจหรือไม่

❑ 1. หายใจทางจมูก ❑ 2. กลั้นหายใจ ❑ 3. หายใจทางปาก ❑ 4. อื่น ๆ ....

9. ยี่ห้ออุปกรณ์ที่คุณเคยใช้ ………………………………………

10. ใช้อุปกรณ์นี้ไปได้นานเท่าไร อุปกรณ์จึงเสีย ☐☐ ปี ☐☐ เดือน

11. คุณทำความสะอาดอุปกรณ์นี้อย่างไร ❑ ล้างน้ำสบู่ ❑ ล้างน้ำเกลือ ❑ ล้างน้ำอุ่น ❑ ต้ม ❑ อื่น ๆ…………..

12. คุณได้อุปกรณ์นี้มาจากที่ใด ❑ โรงพยาบาล ❑ ร้านขายยา ❑ คลินิก

<u>คำชี้แจง</u>

วงกลม ◯ ล้อมรอบตัวเลข ในช่องด้านขวามือที่ท่านพิจารณาว่าเป็นข้อความที่ตรงกับความรู้สึกในขณะนี้

**ความมีประโยชน์ทางคลินิกของอุปกรณ์ล้างจมูก**	**ไม่เห็นด้วยที่สุด** **0**	**เห็นด้วยที่สุด** **10**
1.คุณรู้สึกว่าอุปกรณ์นี้ช่วยให้อาการทางจมูกโดยรวมของคุณลดลง	0 1 2 3 4 5 6 7 8 9 10	
2.คุณรู้สึกว่าอุปกรณ์นี้ช่วยให้อาการคัดจมูกของคุณลดลง	0 1 2 3 4 5 6 7 8 9 10	❑ ไม่มีอาการคัดจมูกมาก่อน
3.คุณรู้สึกว่าอุปกรณ์นี้ช่วยให้อาการน้ำมูกไหลของคุณลดลง	0 1 2 3 4 5 6 7 8 9 10	❑ ไม่มีอาการน้ำมูกไหลมาก่อน
4.คุณรู้สึกว่าอุปกรณ์นี้ช่วยให้คุณสั่งน้ำมูกลดลง	0 1 2 3 4 5 6 7 8 9 10	❑ ไม่ต้องสั่งน้ำมูกมาก่อน
5.คุณรู้สึกว่าอุปกรณ์นี้ช่วยทำให้น้ำมูกข้นน้อยลง	0 1 2 3 4 5 6 7 8 9 10	❑ ไม่มีน้ำมูกข้นมาก่อน

6.คุณรู้สึกว่าอุปกรณ์นี้ช่วยให้อาการปวดบริเวณใบหน้าน้อยลง	0 1 2 3 4 5 6 7 8 9 10	❑ ไม่มีปวดใบหน้ามาก่อน
7คุณรู้สึกว่าอุปกรณ์นี้ช่วยให้น้ำมูกไหลลงคอลดลง	0 1 2 3 4 5 6 7 8 9 10	❑ ไม่มีน้ำมูกไหลลงคอมาก่อน
8.คุณรู้สึกว่าอุปกรณ์นี้ช่วยให้การได้กลิ่นและการรับรสดีขึ้น	0 1 2 3 4 5 6 7 8 9 10	❑ ไม่มีการได้กลิ่นและรับรสที่ผิดปกติมาก่อน
9.คุณรู้สึกว่าอุปกรณ์นี้ช่วยให้อาการจามลดลง	0 1 2 3 4 5 6 7 8 9 10	❑ ไม่มีอาการจามมาก่อน
10.คุณรู้สึกว่าอุปกรณ์นี้ช่วยให้อาการไอลดลง	0 1 2 3 4 5 6 7 8 9 10	❑ ไม่มีอาการไอมาก่อน
11.คุณรู้สึกว่าอุปกรณ์นี้เข้าไปชะล้างน้ำมูกได้ทั่วถึง	0 1 2 3 4 5 6 7 8 9 10	❑ ไม่มีน้ำมูกมาก่อน
12.คุณรู้สึกว่าอุปกรณ์นี้ช่วยให้การนอนโดยรวมของคุณดีขึ้น	0 1 2 3 4 5 6 7 8 9 10	❑ ไม่มีการนอนที่ผิดปกติมาก่อน
**ความสะดวกในการใช้งาน**		
13.คุณรู้สึกว่าอุปกรณ์นี้ใช้งานง่าย	0 1 2 3 4 5 6 7 8 9 10	
14.คุณสามารถใช้งานอุปกรณ์ล้างจมูกนี้ได้โดยที่ไม่ต้องมีคำแนะนำวิธีใช้เขียนกำกับไว้	0 1 2 3 4 5 6 7 8 9 10	
**การเรียนรู้วิธีใช้**		
15.คุณสามารถเรียนรู้วิธีการใช้อุปกรณ์ล้างจมูกนี้ได้อย่างรวดเร็ว	0 1 2 3 4 5 6 7 8 9 10	
16.คุณสามารถจำวิธีการใช้อุปกรณ์ล้างจมูกได้	0 1 2 3 4 5 6 7 8 9 10	
**ความพึงพอใจ**		
17.คุณมีความพึงพอใจในการใช้อุปกรณ์ล้างจมูกชนิดนี้	0 1 2 3 4 5 6 7 8 9 10	
18.คุณจะแนะนำอุปกรณ์ล้างจมูกที่คุณใช้อยู่นี้ให้แก่เพื่อนของคุณ	0 1 2 3 4 5 6 7 8 9 10	

แบบสอบถามที่ 5 ลูกยางล้างจมูก

1. คุณเคยใช้อุปกรณ์นี้ในการรักษาโรคใด

❑ 1. จมูกอักเสบจากภูมิแพ้ ❑ 2. ไซนัสอักเสบเฉียบพลัน ❑ 3. ไซนัสอักเสบเรื้อรัง

❑ 4. หลังผ่าตัด ❑ 5. ไข้หวัด ❑ 6. อื่น ๆ โปรดระบุ.......................................

2. คุณใช้อุปกรณ์นี้มานาน ☐☐ ปี ☐☐ เดือน

3. ความถี่ในการใช้ล้างจมูกโดยประมาณ ☐☐ ครั้ง/วัน หรือ ☐☐ ครั้ง/สัปดาห์

4. คุณล้างจมูกบ่อยแค่ไหน (โดยประมาณ) ❑ 1. ล้างทุกวัน ❑ 2. ล้างเมื่อมีอาการ ❑3.อื่น ๆ ....

5. ใครเป็นผู้สอนคุณล้างจมูก ❑ 1. แพทย์ ❑ 2. พยาบาล ❑ 3. เภสัชกร ❑ 4. ผู้ปกครอง ❑ 5. เรียนรู้เอง

6. ปัญหาของการใช้อุปกรณ์นี้ (ตอบได้มากกว่า 1 ข้อ)

❑ 0. ไม่มี ❑ 1. เจ็บจมูก ❑ 2. น้ำเกลือค้างในจมูก ❑ 3. เลือดกำเดาไหล ❑ 4. ปวดศีรษะ

❑ 5.สำลัก ❑ 6. หูอื้อ ❑ 7. เค็มคอ ❑ 8. อื่น ๆ โปรดระบุ……………………..

7. ท่าที่ใช้ในการล้างจมูก ❑ 1. ก้มหน้า ❑ 2. เงยหน้า ❑ 3. อื่น ๆ…………………………………

8. ระหว่างล้างน้ำเกลือ (น้ำเกลือเข้าจมูก) คุณหายใจหรือไม่

❑ 1. หายใจทางจมูก ❑ 2. กลั้นหายใจ ❑ 3. หายใจทางปาก ❑ 4. อื่น ๆ ....

9. ยี่ห้ออุปกรณ์ที่คุณเคยใช้ ……………………………………….

10. ใช้อุปกรณ์นี้ไปได้นานเท่าไร อุปกรณ์จึงเสีย ☐☐ ปี ☐☐ เดือน

11. คุณทำความสะอาดอุปกรณ์นี้อย่างไร ❑ ล้างน้ำสบู่ ❑ ล้างน้ำเกลือ ❑ ล้างน้ำอุ่น ❑ ต้ม ❑ อื่น ๆ…………..

12. คุณได้อุปกรณ์นี้มาจากที่ใด ❑ โรงพยาบาล ❑ ร้านขายยา ❑ คลินิก

<u>คำชี้แจง</u>

วงกลม ◯ ล้อมรอบตัวเลข ในช่องด้านขวามือที่ท่านพิจารณาว่าเป็นข้อความที่ตรงกับความรู้สึกในขณะนี้

**ความมีประโยชน์ทางคลินิกของอุปกรณ์ล้างจมูก**	**ไม่เห็นด้วยที่สุด** **0**	**เห็นด้วยที่สุด** **10**
1.คุณรู้สึกว่าอุปกรณ์นี้ช่วยให้อาการทางจมูกโดยรวมของคุณลดลง	0 1 2 3 4 5 6 7 8 9 10	
2.คุณรู้สึกว่าอุปกรณ์นี้ช่วยให้อาการคัดจมูกของคุณลดลง	0 1 2 3 4 5 6 7 8 9 10	❑ ไม่มีอาการคัดจมูกมาก่อน
3.คุณรู้สึกว่าอุปกรณ์นี้ช่วยให้อาการน้ำมูกไหลของคุณลดลง	0 1 2 3 4 5 6 7 8 9 10	❑ ไม่มีอาการน้ำมูกไหลมาก่อน
4.คุณรู้สึกว่าอุปกรณ์นี้ช่วยให้คุณสั่งน้ำมูกลดลง	0 1 2 3 4 5 6 7 8 9 10	❑ ไม่ต้องสั่งน้ำมูกมาก่อน
5.คุณรู้สึกว่าอุปกรณ์นี้ช่วยทำให้น้ำมูกข้นน้อยลง	0 1 2 3 4 5 6 7 8 9 10	❑ ไม่มีน้ำมูกข้นมาก่อน

6.คุณรู้สึกว่าอุปกรณ์นี้ช่วยให้อาการปวดบริเวณใบหน้าน้อยลง	0 1 2 3 4 5 6 7 8 9 10	❑ ไม่มีปวดใบหน้ามาก่อน
7คุณรู้สึกว่าอุปกรณ์นี้ช่วยให้น้ำมูกไหลลงคอลดลง	0 1 2 3 4 5 6 7 8 9 10	❑ ไม่มีน้ำมูกไหลลงคอมาก่อน
8.คุณรู้สึกว่าอุปกรณ์นี้ช่วยให้การได้กลิ่นและการรับรสดีขึ้น	0 1 2 3 4 5 6 7 8 9 10	❑ ไม่มีการได้กลิ่นและรับรสที่ผิดปกติมาก่อน
9.คุณรู้สึกว่าอุปกรณ์นี้ช่วยให้อาการจามลดลง	0 1 2 3 4 5 6 7 8 9 10	❑ ไม่มีอาการจามมาก่อน
10.คุณรู้สึกว่าอุปกรณ์นี้ช่วยให้อาการไอลดลง	0 1 2 3 4 5 6 7 8 9 10	❑ ไม่มีอาการไอมาก่อน
11.คุณรู้สึกว่าอุปกรณ์นี้เข้าไปชะล้างน้ำมูกได้ทั่วถึง	0 1 2 3 4 5 6 7 8 9 10	❑ ไม่มีน้ำมูกมาก่อน
12.คุณรู้สึกว่าอุปกรณ์นี้ช่วยให้การนอนโดยรวมของคุณดีขึ้น	0 1 2 3 4 5 6 7 8 9 10	❑ ไม่มีการนอนที่ผิดปกติมาก่อน
**ความสะดวกในการใช้งาน**		
13.คุณรู้สึกว่าอุปกรณ์นี้ใช้งานง่าย	0 1 2 3 4 5 6 7 8 9 10	
14.คุณสามารถใช้งานอุปกรณ์ล้างจมูกนี้ได้โดยที่ไม่ต้องมีคำแนะนำวิธีใช้เขียนกำกับไว้	0 1 2 3 4 5 6 7 8 9 10	
**การเรียนรู้วิธีใช้**		
15.คุณสามารถเรียนรู้วิธีการใช้อุปกรณ์ล้างจมูกนี้ได้อย่างรวดเร็ว	0 1 2 3 4 5 6 7 8 9 10	
16.คุณสามารถจำวิธีการใช้อุปกรณ์ล้างจมูกได้	0 1 2 3 4 5 6 7 8 9 10	
**ความพึงพอใจ**		
17.คุณมีความพึงพอใจในการใช้อุปกรณ์ล้างจมูกชนิดนี้	0 1 2 3 4 5 6 7 8 9 10	
18.คุณจะแนะนำอุปกรณ์ล้างจมูกที่คุณใช้อยู่นี้ให้แก่เพื่อนของคุณ	0 1 2 3 4 5 6 7 8 9 10	

แบบสอบถามที่ 6 สเปรย์ล้างจมูก

1. คุณเคยใช้อุปกรณ์นี้ในการรักษาโรคใด

❑ 1. จมูกอักเสบจากภูมิแพ้ ❑ 2. ไซนัสอักเสบเฉียบพลัน ❑ 3. ไซนัสอักเสบเรื้อรัง

❑ 4. หลังผ่าตัด ❑ 5. ไข้หวัด ❑ 6. อื่น ๆ โปรดระบุ.......................................

2. คุณใช้อุปกรณ์นี้มานาน ☐☐ ปี ☐☐ เดือน

3. ความถี่ในการใช้ล้างจมูกโดยประมาณ☐☐ ครั้ง/วัน หรือ ☐☐ ครั้ง/สัปดาห์

4. คุณล้างจมูกบ่อยแค่ไหน (โดยประมาณ) ❑ 1. ล้างทุกวัน ❑ 2. ล้างเมื่อมีอาการ ❑3.อื่น ๆ ....

5. ใครเป็นผู้สอนคุณล้างจมูก ❑ 1. แพทย์ ❑ 2. พยาบาล ❑ 3. เภสัชกร ❑ 4. ผู้ปกครอง ❑ 5. เรียนรู้เอง

6. ปัญหาของการใช้อุปกรณ์นี้ (ตอบได้มากกว่า 1 ข้อ)

❑ 0. ไม่มี ❑ 1. เจ็บจมูก ❑ 2. น้ำเกลือค้างในจมูก❑ 3. เลือดกำเดาไหล ❑ 4. ปวดศีรษะ

❑ 5.สำลัก ❑ 6. หูอื้อ ❑ 7. เค็มคอ ❑ 8. อื่น ๆ โปรดระบุ……………………..

7. ท่าที่ใช้ในการล้างจมูก ❑ 1. ก้มหน้า ❑ 2. เงยหน้า ❑ 3. อื่น ๆ…………………………………

8. ระหว่างล้างน้ำเกลือ (น้ำเกลือเข้าจมูก) คุณหายใจหรือไม่

❑ 1.หายใจทางจมูก ❑ 2. กลั้นหายใจ ❑ 3. หายใจทางปาก ❑ 4. อื่น ๆ ....

9. ยี่ห้ออุปกรณ์ที่คุณเคยใช้ ………………………………………..

10. ใช้อุปกรณ์นี้ไปได้นานเท่าไร อุปกรณ์จึงเสีย ☐☐ ปี ☐☐ เดือน

11. คุณทำความสะอาดอุปกรณ์นี้อย่างไร ❑ ล้างน้ำสบู่ ❑ ล้างน้ำเกลือ ❑ ล้างน้ำอุ่น ❑ ต้ม ❑ อื่น ๆ…………..

12. คุณได้อุปกรณ์นี้มาจากที่ใด ❑ โรงพยาบาล ❑ ร้านขายยา ❑ คลินิก

คำชี้แจง

วงกลม ○ ล้อมรอบตัวเลข ในช่องด้านขวามือที่ท่านพิจารณาว่าเป็นข้อความที่ตรงกับความรู้สึกในขณะนี้

**ความมีประโยชน์ทางคลินิกของอุปกรณ์ล้างจมูก**	**ไม่เห็นด้วยที่สุด** **0** — **เห็นด้วยที่สุด** **10**	
1.คุณรู้สึกว่าอุปกรณ์นี้ช่วยให้อาการทางจมูกโดยรวมของคุณลดลง	0 1 2 3 4 5 6 7 8 9 10	
2.คุณรู้สึกว่าอุปกรณ์นี้ช่วยให้อาการคัดจมูกของคุณลดลง	0 1 2 3 4 5 6 7 8 9 10	❑ ไม่มีอาการคัดจมูกมาก่อน
3.คุณรู้สึกว่าอุปกรณ์นี้ช่วยให้อาการน้ำมูกไหลของคุณลดลง	0 1 2 3 4 5 6 7 8 9 10	❑ ไม่มีอาการน้ำมูกไหลมาก่อน
4.คุณรู้สึกว่าอุปกรณ์นี้ช่วยให้คุณสั่งน้ำมูกลดลง	0 1 2 3 4 5 6 7 8 9 10	❑ ไม่ต้องสั่งน้ำมูกมาก่อน
5.คุณรู้สึกว่าอุปกรณ์นี้ช่วยทำให้น้ำมูกข้นน้อยลง	0 1 2 3 4 5 6 7 8 9 10	❑ ไม่มีน้ำมูกข้นมาก่อน

6.คุณรู้สึกว่าอุปกรณ์นี้ช่วยให้อาการปวดบริเวณใบหน้าน้อยลง	0 1 2 3 4 5 6 7 8 9 10	❑ ไม่มีปวดใบหน้ามาก่อน
7คุณรู้สึกว่าอุปกรณ์นี้ช่วยให้น้ำมูกไหลลงคอลดลง	0 1 2 3 4 5 6 7 8 9 10	❑ ไม่มีน้ำมูกไหลลงคอมาก่อน
8.คุณรู้สึกว่าอุปกรณ์นี้ช่วยให้การได้กลิ่นและการรับรสดีขึ้น	0 1 2 3 4 5 6 7 8 9 10	❑ ไม่มีการได้กลิ่นและรับรสที่ผิดปกติมาก่อน
9.คุณรู้สึกว่าอุปกรณ์นี้ช่วยให้อาการจามลดลง	0 1 2 3 4 5 6 7 8 9 10	❑ ไม่มีอาการจามมาก่อน
10.คุณรู้สึกว่าอุปกรณ์นี้ช่วยให้อาการไอลดลง	0 1 2 3 4 5 6 7 8 9 10	❑ ไม่มีอาการไอมาก่อน
11.คุณรู้สึกว่าอุปกรณ์นี้เข้าไปชะล้างน้ำมูกได้ทั่วถึง	0 1 2 3 4 5 6 7 8 9 10	❑ ไม่มีน้ำมูกมาก่อน
12.คุณรู้สึกว่าอุปกรณ์นี้ช่วยให้การนอนโดยรวมของคุณดีขึ้น	0 1 2 3 4 5 6 7 8 9 10	❑ ไม่มีการนอนที่ผิดปกติมาก่อน
**ความสะดวกในการใช้งาน**		
13.คุณรู้สึกว่าอุปกรณ์นี้ใช้งานง่าย	0 1 2 3 4 5 6 7 8 9 10	
14.คุณสามารถใช้งานอุปกรณ์ล้างจมูกนี้ได้โดยที่ไม่ต้องมีคำแนะนำวิธีใช้เขียนกำกับไว้	0 1 2 3 4 5 6 7 8 9 10	
**การเรียนรู้วิธีใช้**		
15.คุณสามารถเรียนรู้วิธีการใช้อุปกรณ์ล้างจมูกนี้ได้อย่างรวดเร็ว	0 1 2 3 4 5 6 7 8 9 10	
16.คุณสามารถจำวิธีการใช้อุปกรณ์ล้างจมูกได้	0 1 2 3 4 5 6 7 8 9 10	
**ความพึงพอใจ**		
17.คุณมีความพึงพอใจในการใช้อุปกรณ์ล้างจมูกชนิดนี้	0 1 2 3 4 5 6 7 8 9 10	
18.คุณจะแนะนำอุปกรณ์ล้างจมูกที่คุณใช้อยู่นี้ให้แก่เพื่อนของคุณ	0 1 2 3 4 5 6 7 8 9 10	

แบบสอบถามที่ 7 ที่หยดล้างจมูก

1. คุณเคยใช้อุปกรณ์นี้ในการรักษาโรคใด

❑ 1. จมูกอักเสบจากภูมิแพ้ ❑ 2. ไซนัสอักเสบเฉียบพลัน ❑ 3. ไซนัสอักเสบเรื้อรัง

❑ 4. หลังผ่าตัด ❑ 5. ไข้หวัด ❑ 6. อื่น ๆ โปรดระบุ.......................................

2. คุณใช้อุปกรณ์นี้มานาน ☐☐ ปี ☐☐ เดือน

3. ความถี่ในการใช้ล้างจมูกโดยประมาณ ☐☐ ครั้ง/วัน หรือ ☐☐ ครั้ง/สัปดาห์

4. คุณล้างจมูกบ่อยแค่ไหน (โดยประมาณ) ❑ 1. ล้างทุกวัน ❑ 2. ล้างเมื่อมีอาการ ❑3.อื่น ๆ ....

5. ใครเป็นผู้สอนคุณล้างจมูก ❑ 1. แพทย์ ❑ 2. พยาบาล ❑ 3. เภสัชกร ❑ 4. ผู้ปกครอง ❑ 5. เรียนรู้เอง

6. ปัญหาของการใช้อุปกรณ์นี้ (ตอบได้มากกว่า 1 ข้อ)

❑ 0. ไม่มี ❑ 1. เจ็บจมูก ❑ 2. น้ำเกลือค้างในจมูก ❑ 3. เลือดกำเดาไหล ❑ 4. ปวดศีรษะ

❑ 5.สำลัก ❑ 6. หูอื้อ ❑ 7. เค็มคอ ❑ 8. อื่น ๆ โปรดระบุ……………………..

7. ท่าที่ใช้ในการล้างจมูก ❑ 1. ก้มหน้า ❑ 2. เงยหน้า ❑ 3. อื่น ๆ…………………………………

8. ระหว่างล้างน้ำเกลือ (น้ำเกลือเข้าจมูก) คุณหายใจหรือไม่

❑ 1. หายใจทางจมูก ❑ 2. กลั้นหายใจ ❑ 3. หายใจทางปาก ❑ 4. อื่น ๆ ....

9. ยี่ห้ออุปกรณ์ที่คุณเคยใช้ ………………………………………

10. ใช้อุปกรณ์นี้ไปได้นานเท่าไร อุปกรณ์จึงเสีย ☐☐ ปี ☐☐ เดือน

11. คุณทำความสะอาดอุปกรณ์นี้อย่างไร ❑ ล้างน้ำสบู่ ❑ ล้างน้ำเกลือ ❑ ล้างน้ำอุ่น ❑ ต้ม ❑ อื่น ๆ…………..

12. คุณได้อุปกรณ์นี้มาจากที่ใด ❑ โรงพยาบาล ❑ ร้านขายยา ❑ คลินิก

คำชี้แจง

วงกลม ○ ล้อมรอบตัวเลข ในช่องด้านขวามือที่ท่านพิจารณาว่าเป็นข้อความที่ตรงกับความรู้สึกในขณะนี้

**ความมีประโยชน์ทางคลินิกของอุปกรณ์ล้างจมูก**	**ไม่เห็นด้วยที่สุด** **0** — **เห็นด้วยที่สุด** **10**	
1.คุณรู้สึกว่าอุปกรณ์นี้ช่วยให้อาการทางจมูกโดยรวมของคุณลดลง	0 1 2 3 4 5 6 7 8 9 10	
2.คุณรู้สึกว่าอุปกรณ์นี้ช่วยให้อาการคัดจมูกของคุณลดลง	0 1 2 3 4 5 6 7 8 9 10	❑ ไม่มีอาการคัดจมูกมาก่อน
3.คุณรู้สึกว่าอุปกรณ์นี้ช่วยให้อาการน้ำมูกไหลของคุณลดลง	0 1 2 3 4 5 6 7 8 9 10	❑ ไม่มีอาการน้ำมูกไหลมาก่อน
4.คุณรู้สึกว่าอุปกรณ์นี้ช่วยให้คุณสั่งน้ำมูกลดลง	0 1 2 3 4 5 6 7 8 9 10	❑ ไม่ต้องสั่งน้ำมูกมาก่อน
5.คุณรู้สึกว่าอุปกรณ์นี้ช่วยทำให้น้ำมูกข้นน้อยลง	0 1 2 3 4 5 6 7 8 9 10	❑ ไม่มีน้ำมูกข้นมาก่อน

6.คุณรู้สึกว่าอุปกรณ์นี้ช่วยให้อาการปวดบริเวณใบหน้าน้อยลง	0 1 2 3 4 5 6 7 8 9 10	❑ ไม่มีปวดใบหน้ามาก่อน
7คุณรู้สึกว่าอุปกรณ์นี้ช่วยให้น้ำมูกไหลลงคอลดลง	0 1 2 3 4 5 6 7 8 9 10	❑ ไม่มีน้ำมูกไหลลงคอมาก่อน
8.คุณรู้สึกว่าอุปกรณ์นี้ช่วยให้การได้กลิ่นและการรับรสดีขึ้น	0 1 2 3 4 5 6 7 8 9 10	❑ ไม่มีการได้กลิ่นและรับรสที่ผิดปกติมาก่อน
9.คุณรู้สึกว่าอุปกรณ์นี้ช่วยให้อาการจามลดลง	0 1 2 3 4 5 6 7 8 9 10	❑ ไม่มีอาการจามมาก่อน
10.คุณรู้สึกว่าอุปกรณ์นี้ช่วยให้อาการไอลดลง	0 1 2 3 4 5 6 7 8 9 10	❑ ไม่มีอาการไอมาก่อน
11.คุณรู้สึกว่าอุปกรณ์นี้เข้าไปชะล้างน้ำมูกได้ทั่วถึง	0 1 2 3 4 5 6 7 8 9 10	❑ ไม่มีน้ำมูกมาก่อน
12.คุณรู้สึกว่าอุปกรณ์นี้ช่วยให้การนอนโดยรวมของคุณดีขึ้น	0 1 2 3 4 5 6 7 8 9 10	❑ ไม่มีการนอนที่ผิดปกติมาก่อน
**ความสะดวกในการใช้งาน**		
13.คุณรู้สึกว่าอุปกรณ์นี้ใช้งานง่าย	0 1 2 3 4 5 6 7 8 9 10	
14.คุณสามารถใช้งานอุปกรณ์ล้างจมูกนี้ได้โดยที่ไม่ต้องมีคำแนะนำวิธีใช้เขียนกำกับไว้	0 1 2 3 4 5 6 7 8 9 10	
**การเรียนรู้วิธีใช้**		
15.คุณสามารถเรียนรู้วิธีการใช้อุปกรณ์ล้างจมูกนี้ได้อย่างรวดเร็ว	0 1 2 3 4 5 6 7 8 9 10	
16.คุณสามารถจำวิธีการใช้อุปกรณ์ล้างจมูกได้	0 1 2 3 4 5 6 7 8 9 10	
**ความพึงพอใจ**		
17.คุณมีความพึงพอใจในการใช้อุปกรณ์ล้างจมูกชนิดนี้	0 1 2 3 4 5 6 7 8 9 10	
18.คุณจะแนะนำอุปกรณ์ล้างจมูกที่คุณใช้อยู่นี้ให้แก่เพื่อนของคุณ	0 1 2 3 4 5 6 7 8 9 10	
