# Supplementary material for: Effectiveness of nasal irrigation devices: a Thai multicentre survey
Source: PeerJ. 2019 May 27;7:e7000. doi: 10.7717/peerj.7000 (PMC6542345; doi:10.7717/peerj.7000)
Supplement: Supplemental Information 4 [file peerj-07-7000-s004.pdf]

ผู้ตอบแบบสอบถามหมายเลข ____________

<u>แบบสอบถามสำหรับแพทย์</u>

1. ในปัจจุบันคุณได้สั่งใช้อุปกรณ์ล้างจมูกชนิดใดบ้าง (ตอบได้มากกว่า 1 ข้อ)

❑ หลอดฉีดยา ❑หลอดฉีดยามีจุก ❑ ขวดล้างจมูกแบบบีบ

❑ขวดล้างจมูกแบบเท บีบไม่ได้ ❑ ลูกยางล้างจมูก ❑ สเปรย์ล้างจมูก

❑ที่หยดล้างจมูก

2. คุณสั่งใช้อุปกรณ์ล้างจมูกในกรณีใดบ้าง (ตอบได้มากกว่า 1 ข้อ)

❑ จมูกอักเสบจากภูมิแพ้ ❑ ไซนัสอักเสบเฉียบพลัน ❑ ไซนัสอักเสบเรื้อรัง

❑ หลังผ่าตัด ❑ไข้หวัด ❑ อื่น ๆ โปรดระบุ……………………..

3. ข้อดี ข้อเสีย ของอุปกรณ์ที่สั่งใช้

3.1 หลอดฉีดยา

-ข้อดี ………………………………………………………………………………………

-ข้อเสีย ……………………………………………………………………………………

3.2 หลอดฉีดยามีจุก

-ข้อดี ………………………………………………………………………………………

-ข้อเสีย ……………………………………………………………………………………

3.3 ขวดล้างจมูกแบบบีบ

-ข้อดี ………………………………………………………………………………………

-ข้อเสีย ……………………………………………………………………………………

3.4 ขวดล้างจมูกแบบเท บีบไม่ได้

-ข้อดี ………………………………………………………………………………………

-ข้อเสีย ……………………………………………………………………………………

3.5 ลูกยางล้างจมูก

-ข้อดี ………………………………………………………………………………………

-ข้อเสีย ……………………………………………………………………………………

3.6 สเปรย์ล้างจมูก

-ข้อดี ………………………………………………………………………………………

-ข้อเสีย ……………………………………………………………………………………

4. ชนิดน้ำเกลือที่คุณสั่งใช้ (ตอบได้มากกว่า 1 ข้อ)

❑ Hypotonic solution ❑ Isotonic solution ❑ Hypertonic solution

สาเหตุที่สั่งใช้…………………………………………………………………………….....

5. คุณจะ<u>ไม่</u>สั่งใช้อุปกรณ์ล้างจมูกต่อไปนี้ (ตอบได้หลายชนิด)

- <u>ไม่</u>สั่งใช้ชนิด……………………..เพราะ…………………………….……………………...
- <u>ไม่</u>สั่งใช้ชนิด……………………..เพราะ…………………………….……………………...
- <u>ไม่</u>สั่งใช้ชนิด……………………..เพราะ…………………………….……………………...

ผู้ตอบแบบสอบถามหมายเลข ______________

6. คุณได้อธิบายวิธีการใช้อุปกรณ์ให้ผู้ป่วยทราบหรือไม่

❑ อธิบาย

❑ ไม่ได้อธิบาย เพราะ..
